# Colombeau products of distributions

**DOI:** 10.1186/s40064-016-3742-8

**Published:** 2016-11-29

**Authors:** Marija Miteva, Biljana Jolevska-Tuneska, Tatjana Atanasova-Pacemska

**Affiliations:** 1 Faculty of Computer Science, University Goce Delcev-Stip, Stip, Republic of Macedonia; 2 Faculty of Electrical Engineering and Information Technologies, Ss. Cyril and Methodius University, Skopje, Republic of Macedonia

**Keywords:** Distributions, Colombeau algebra, Colombeau generalized functions, Multiplication of distributions

## Abstract

In this paper, some products of distributions are derived. The results are obtained in Colombeau algebra of generalized functions, which is the most relevant algebraic construction for dealing with Schwartz distributions. Colombeau algebra ${\mathcal{G}}({\mathbf{R}})$ contains the space ${\mathcal{D}'}({\mathbf{R}})$ of Schwartz distributions as a subspace, and has a notion of 'association’ that allows us to evaluate the results in terms of distributions.

## Background

The large employment of distributions in different mathematical fields and other natural sciences has imposed the need for solving two main problems that distribution theory comes across: multiplication of distributions (no two distributions can always be multiplied) and differentiating the product of distributions (the product of distributions does not always satisfy the Leibniz rule). Therefore, many attempts have been made to define the product of distributions (Fisher 1971, 1980; Zhi and Fisher 1989), or rather to enlarge the number of existing products. Many attempts have also been made to include the distributions in differential algebras (Oberguggenberger and Todorov 1998).

According to the distribution theory (Zemanian 1965; Gelfand and Shilov 1964), we can distinguish two complementary points of view:

The first one is that distribution can be considered as a continuous linear functional *f* acting on a smooth function $\varphi$ with compact support, i.e., we have a linear map $\varphi \rightarrow \left\langle {f,\,\varphi } \right\rangle$ where $\varphi$ is called *test function*.

The second one is the sequential approach: taking a sequence of smooth functions $({{\varphi_n}})$ converging to the Dirac $\delta$ function, we obtain a family of regularization $(f_n)$ by the convolution product

1 $${f_n}\left( x \right) = \left({f * {\varphi_n}} \right) \left( x \right) = \left\langle {f\left( y \right) ,\,{\varphi_n}\left({x - y} \right) } \right\rangle$$

which converges weakly to the distribution *f*. We identify all the sequences that converge weakly to the same limit, and consider them as an equivalence class. The elements of each equivalence class are called *representatives* of the appropriate distribution *f*. This way we obtain sequential representation of distributions. Some authors use equivalence classes of nets of regularization, i.e. the $\delta$-net ${\left({{\varphi_\varepsilon }} \right)_{\varepsilon > 0}}$ defined with ${\varphi_\varepsilon } = \frac{1}{\varepsilon }\varphi \left({\frac{x}{\varepsilon }} \right)$.

By the regularization process, the nonlinear structure is lost in a way by identifying sequences with their limit. Actually, all the operations then are done on the regularized functions (sequences of smooth functions) and with the inverse process starting from the result, the function is returned from the regularization. So, we have to get nonlinear theory of generalized functions that will work with regularization.

The optimal solution for overcoming the problems that Schwartz theory of distributions is concerned with was offered by Colombeau (1984, 1985). He constructed an associative differential algebra of generalized functions ${\mathcal{G}}\left({\mathbf{R}}\right)$, which contains the space ${\mathcal{D}'}\left({\mathbf{R}}\right)$ of distributions as subspace and the algebra of ${C^\infty }$—functions as subalgebra. This theory of generalized functions of Colombeau actually generalizes the theory of Schwartz distributions: these new Colombeau generalized functions can be differentiated in the same way as distributions, but where multiplication and other nonlinear operations are concerned, it is significant that the result of these operations always exists in this algebra as Colombeau generalized function [How Colombeau algebra ${\mathcal{G}}$ can be used for treating linear and nonlinear problems, including singularities, one can see in Jolevska et al. (2007)]. These new generalized functions are very much related to the distributions, in the sense that their definition may be considered as a natural evolution of the Schwartz definition of distributions.

The notion of 'association’ in ${\mathcal{G}}$ is a faithful generalization of the equality of distributions, and again enables us to interpret results in terms of distributions.

Due to all these properties, Colombeau theory has found extensive application in different natural sciences and engineering, especially in fields where products of distributions with coinciding singularities are considered. Such products are, for example, products that include Dirac delta function $\delta$ which has singular point support.

Before distribution theory come to be introduced, Dirac delta function, as with many other concepts in physics and engineering, was heuristically understood with properties given to coincide with experimental results and was considered adequate for solving complicated problems. Delta function $\delta$ was well defined to represent certain types of infinity concentrated at a single point (by the physicists $\delta$ was used to represent the charge density of a point particle—the charge is concentrated in a single point, i.e., finite amount of charge is packed into zero volume, thus the charge density must be infinite at that single point, and its derivative $\delta'$ was used to represent a dipole of unit electric moment at the origin). After the theory of distributions was invented (in the early 1950s), the mathematical meaning of these concepts has been established and delta function with the same properties is considered as distribution. But, the problem still occurs when multiplying two distributions in the Schwartz’s space (for example, ${\delta ^2}$ doesn’t exist in this space). As we said above, Colombeau algebra was constructed in a way that many problems with multiplication of distributions could be avoided. About applications of Colombeau theory of generalized functions, one can read papers (Aragona et al. 2014; Gsponer 2009; Ohkitani and Dowker 2010; Prusa and Rajagopal 2016; Steinbauer and Vickers 2006; Capar 2013; Nigsch and Samman 2013; Steinbauer 1997; Alimohammady 2014; Sojanovic 2013; Farassat 1994). As we can see in many of these papers, products of delta function and its derivatives with other distributions with singularities, but with continuous functions too, appear while solving various problems in physics and engineering.

In this paper we obtain some products that include derivatives of delta function, in Colombeau algebra, in terms of associated distributions. Other products of distributions, evaluated in the same way, can be found in Damyanov (1997, 2005, 2006), Miteva and Jolevska (2012), Jolevska and Atanasova (2013) and Miteva et al. (2014). The results can be reformulated as regularized products in the classical distribution theory.

## Colombeau algebra

In this section we will give notations and definitions from Colombeau theory that we have used while evaluating the main results.

${\mathbf{N_0}}$ is the set of non-negative integers, i.e. ${\mathbf{N_0}} = {\mathbf{N}}\cup \left\{ 0 \right\}$.

Let ${{\mathcal{D}}\left({\mathbf{R}} \right) }$ be the space of all smooth functions $\varphi :{\mathbf{R}} \rightarrow {\mathbf{C}}$ with compact support.

For $q \in {\mathbf{N_0}}$ we denote

2 $${A_q}\left({\mathbf{R}} \right) = \left\{ {\varphi \left( x \right) \in {\mathcal{D}}\left({\mathbf{R}} \right) \left| {\int \limits_{\mathbf{R}} {\varphi \left( x \right) dx} = 1 \; and\; \int \limits_{\mathbf{R}} {{x^j}\varphi \left( x \right) dx = 0,\; j = 1,\ldots ,q} } \right. } \right\}$$

The elements of the set ${A_q}({\mathbf{R}})$ are called *test functions*.

It is obvious that ${A_1} \supset {A_2} \supset {A_3}\ldots$. Colombeau in his books has proved that the sets ${A_k}$ are non empty for all $k \in {\mathbf{N}}$.

For $\varphi \in {A_q}({\mathbf{R}})$ and $\varepsilon > 0$ it is denoted as ${\varphi_\varepsilon} = \frac{1}{\varepsilon}\varphi \left({\frac{x}{\varepsilon}}\right)$ and $\mathop{\varphi}\limits^{\vee} (x) = \varphi({-x})$.

Let ${\mathcal{E}}\left({\mathbf{R}}\right)$ be the algebra of functions $f\left({\varphi , x} \right) :\,{A_0}\left({\mathbf{R}} \right) \times {\mathbf{R}} \rightarrow {\mathbf{C}}$ that are infinitely differentiable for fixed 'parameter’ $\varphi$. The generalized functions of Colombeau are elements of the quotient algebra

3 $${\mathcal{G}}\equiv {\mathcal{G}}\left({\mathbf{R}}\right) = \frac{{{\mathcal{E}}_M\left[ {\mathbf{R}} \right] }}{{{\mathcal{I}}\left[ {\mathbf{R}} \right] }}$$

where ${{{\mathcal{E}}_M\left[ {\mathbf{R}} \right] }}$ is the subalgebra of ‘moderate’ functions such that for each compact subset *K* of ${\mathbf{R}}$ and any $p \in {\mathbf{N_0}}$ there is a $q \in {\mathbf{N}}$, such that for each $\varphi \in {A_q}\left({\mathbf{R}} \right)$ there are $c> 0,\,\eta > 0$ and it holds:

4 $$\mathop{\sup}\limits_{x \in K} \left| {{\partial ^p}f\left({{\varphi_\varepsilon},x} \right) } \right| \le c{\varepsilon^{- q}}$$

for $0< \varepsilon < \eta$ and ${{\mathcal{I}}\left[ {\mathbf{R}}\right] }$ is an ideal of ${{{\mathcal{E}}_M\left[ {\mathbf{R}} \right] }}$ consisting of all functions $f\left({\varphi ,x} \right)$ such that for each compact subset *K* of ${\mathbf{R}}$ and any $p \in {\mathbf{N_0}}$ there is a $q \in {\mathbf{N}}$ such that for every $r \ge q$ and each $\varphi \in {A_r}\left({\mathbf{R}} \right)$ there are $c> 0,\,\eta > 0$ and it holds:

5 $$\mathop {\sup }\limits_{x \in K} \left| {{\partial ^p}f\left({{\varphi_\varepsilon },x} \right) } \right| \le c{\varepsilon^{r - q}}$$

for $$0< \varepsilon < \eta$$.

The distributions on ${\mathbf{R}}$ are embedded in the Colombeau algebra ${\mathcal{G}}\left({\mathbf{R}}\right)$ by the map:

6 $$i:{\mathcal{D}}'\left({\mathbf{R}}\right) \rightarrow {\mathcal{G}}\left({\mathbf{R}}\right) :\,u \rightarrow \widetilde{u} = \left\{ {\widetilde{u}\left({\varphi ,x} \right) = \left({u * \mathop \varphi \limits ^ \vee } \right) \left( x \right) :\varphi \in {A_q}\left({\mathbf{R}} \right) } \right\}$$

where $*$ denotes the convolution product of two distributions and is given by:

7 $$\left({f * g} \right) \left( x \right) = \int \limits_{\mathbf{R}} {f\left( y \right) g\left({x - y} \right) } dy.$$

We should notice that the sequential approach (regularization method) mentioned in the previous section is used here. Thus, an element $f \in {\mathcal{G}}$ (a generalized function of Colombeau) is actually an equivalence class $\left[ f \right] = \left[ {{f_\varepsilon } + \mathcal{I}} \right]$ of an element ${f_\varepsilon } \in {{\mathcal{E}}_M}$ which is called *representative* of *f*. Multiplication and differentiation of generalized functions are performed on arbitrary representatives of the respective generalized functions.

The meaning of the term ‘association’ in ${\mathcal{G}}\left({\mathbf{R}}\right)$ is given with the next two definitions.

### **Definition 1**

Generalized functions $f,g \in {\mathcal{G}}\left({\mathbf{R}}\right)$ are said to be *associated*, denoted $f \approx g$, if for each representative $f\left({{\varphi_\varepsilon },x} \right)$ and $g\left({{\varphi_\varepsilon },x} \right)$ and arbitrary $\psi \left( x \right) \in {{\mathcal{D}}\left({\mathbf{R}} \right) }$ there is a $q \in {\mathbf{N_0}}$ such that for any $\varphi \left( x \right) \in {A_q}\left({\mathbf{R}} \right)$

8 $$\mathop {\lim }\limits_{\varepsilon \rightarrow {0_ + }} \int \limits_{\mathbf{R}} {\left| {f\left({{\varphi_\varepsilon },x} \right) - g\left({{\varphi_\varepsilon },x} \right) } \right| } \psi \left( x \right) dx = 0$$

### **Definition 2**

A generalized function $f \in {\mathcal{G}}$ is said to admit some $u \in {\mathcal{D}}'\left({\mathbf{R}}\right)$ as 'associated distribution’, denoted $f \approx u$, if for each representative $f\left({{\varphi_\varepsilon },x} \right)$ of *f* and any $\psi \left( x \right) \in {{\mathcal{D}}\left({\mathbf{R}} \right) }$ there is a $q \in {\mathbf{N_0}}$ such that for any $\varphi \left( x \right) \in {A_q}\left({\mathbf{R}} \right)$

9 $$\mathop{\lim }\limits_{\varepsilon \rightarrow {0_ + }} \int \limits_{\mathbf{R}}{f\left({{\varphi_\varepsilon },x} \right) } \psi \left( x \right) dx = \left\langle {u,\psi } \right\rangle$$

The representatives chosen in the above two definitions don’t affect the result. The distribution associated, if it exists, is unique and the association is a faithful generalization of the equality of distributions.

If we multiply two distributions embedded in ${\mathcal{G}}$, as a result we always obtain a generalized function of Colombeau. But, it may not always be associated to a third distribution, so if the product of two distributions embedded in Colombeau algebra ${\mathcal{G}}$ admits an associated distribution, we say that *Colombeau product* of those two distributions exists. If the regularized model product of two distributions exists, then their Colombeau product also exists and it is the same as the first one.

## Main results

### **Theorem 1**

*The product of the generalized functions* $\widetilde{\left({\cos x - \sin x} \right) }$ *and* $\widetilde{{\delta ^{\left( r \right) }}\left( x \right) }$ *for* $r=0,1,2,\dots$ *in* ${\mathcal{G}}({\mathbf{R}})$ *admits associated distribution and it holds:*

10 $$\widetilde{\left({\cos x - \sin x} \right) } \cdot \widetilde{{\delta ^{\left( r \right) }}\left( x \right) } \approx \sum \limits_{i = 0}^r {\left( \begin{array}{l} r\\ i \end{array} \right) } {\left({ - 1} \right) ^{{b_i}}}{\delta ^{\left({r - i} \right) }}\left( x \right)$$

*where* ${b_i} = 1 + \left[ {\frac{i}{2}} \right]$ and $\left[ x \right]$ *is the floor function.*

### *Proof*

Using Taylor’s Theorem for the functions $\sin x$ and $\cos x$ we have:

11 $$\cos x - \sin x = \sum \limits_{i = 0}^r {{{\left({ - 1} \right) }^{{a_i}}}} \cdot \frac{{{x^i}}}{{i!}} + {R_r}\left( x \right)$$

where ${a_i} = \left[ {\frac{{i + 1}}{2}} \right]$ and ${R_r}\left( x \right) = {\left({ - 1} \right) ^{{a_{r + 1}}}}\frac{{{x^{r + 1}}}}{{\left({r + 1} \right) !}}\left[ {{{\cos }^{\left({r + 1} \right) }}\left({\eta x} \right) - {{\sin }^{\left({r + 1} \right) }}\left({\eta x} \right) } \right]$ for $0< \eta < 1$.

About the embedding of the function ${\left({\cos x - \sin x} \right) }$ in Colombeau algebra, for any $\varphi \in A_0({\mathbf{R}})$ we have:

12 $$\begin{aligned}\left({\widetilde{\cos x - \sin x}} \right) \left({{\varphi_\varepsilon },x} \right) &= \left[ {\left({\cos x - \sin x} \right) *{{\mathop \varphi \limits ^ \vee }_\varepsilon }} \right] \left( x \right) \\ &= \frac{1}{\varepsilon }\int \limits_{ - \infty }^\infty {\left({\cos y - \sin y} \right) \varphi \left({\frac{{y - x}}{\varepsilon }} \right) } dy \\ &= \frac{1}{\varepsilon }\sum \limits_{i = 0}^r {{{\frac{{\left({ - 1} \right) }}{{i!}}}^{{a_i}}}} \int \limits_{ - \infty }^\infty {{y^i}} \varphi \left({\frac{{y - x}}{\varepsilon }} \right) dy \\ &\quad +\,\frac{1}{\varepsilon }\cdot \int \limits_{ - \infty }^\infty {{R_r}\left( y \right) \varphi \left({\frac{{y - x}}{\varepsilon }} \right) dy} \end{aligned}$$

We suppose that ${\text{supp}}\varphi (x)\subseteq [-l,l]\,,$ without loss of generality. Then using the substitution $\frac{{y - x}}{\varepsilon } = t$ we obtain:

13 $$\begin{aligned}\widetilde{\left({\cos x - \sin x} \right) }\left({{\varphi_\varepsilon },x} \right)&={\sum \limits_{i = 0}^r {\frac{{\left({ - 1} \right) }}{{i!}}} ^{{a_i}}}\,\,\int \limits_{ - l}^l {{{\left({\varepsilon t + x} \right) }^i}} \varphi \left( t \right) dt + \int \limits_{ - l}^l {{R_r}\left({\varepsilon t + x} \right) \varphi \left( t \right) dt} \\ & = {\sum \limits_{i = 0}^r {\frac{{\left({ - 1} \right) }}{{i!}}} ^{{a_i}}}\,\,\int \limits_{ - l}^l {{{\left({\varepsilon t + x} \right) }^i}} \varphi \left( t \right) dt + O\left( \varepsilon \right) \end{aligned}$$

The reminder term is bounded, i.e $O\left( \varepsilon \right)$ due to the properties of the function $\varphi$.

In a similar way we obtain the embedding of the distribution ${\delta ^{\left( r \right) }}\left( x \right)$ in Colombeau algebra:

14 $$\widetilde{\delta ^{(r)}}\left( \varphi_{\varepsilon },x\right) =\frac{(-1)^{r}}{\varepsilon ^{r+1}}\varphi ^{(r)}\left( -\frac{x}{\varepsilon }\right)$$

Then, for any $\psi (x)\in \mathcal{D}({\mathbf{R}})$ we have:

15 $$\begin{aligned}&\left\langle {\left({\widetilde{\cos x - \sin x}} \right) \left({{\varphi_\varepsilon },x} \right) \cdot \widetilde{{\delta ^{\left( r \right) }}}\left({{\varphi_\varepsilon },x} \right) ,\psi \left( x \right) } \right\rangle \\ &\quad = \,\frac{{{{\left({ - 1} \right) }^r}}}{{{\varepsilon ^{r + 1}}}}\int \limits_{ - \infty }^\infty {{{\sum \limits_{i = 0}^r {\frac{{\left({ - 1} \right) }}{{i!}}} }^{{a_i}}}\left({\int \limits_{ - l}^l {{{\left({\varepsilon t + x} \right) }^i}} \varphi \left( t \right) dt} \right) } {\varphi ^{\left( r \right) }}\left({ - \frac{x}{\varepsilon }} \right) \psi \left( x \right) dx + O\left( \varepsilon \right) \\ &\quad = \,\frac{{{{\left({ - 1} \right) }^{r + 1}}}}{{{\varepsilon ^r}}}\sum \limits_{i = 0}^r {{{\frac{{\left({ - 1} \right) }}{{i!}}}^{{a_i}}}} \int \limits_{ - l}^l {{\varphi ^{\left( r \right) }}\left( u \right) \psi \left({ - \varepsilon u} \right) \int \limits_{ - l}^l {{{\left({\varepsilon t - \varepsilon u} \right) }^i}} \varphi \left( t \right) dt} du + O\left( \varepsilon \right) \end{aligned}$$

where we have used substitution $u = - \frac{x}{\varepsilon }$.

Applying Taylor’s Theorem for the function $\psi$ we have:

16 $$\psi \left({ - \varepsilon u} \right) = \sum \limits_{j = 0}^r {\frac{{{\psi ^{\left( j \right) }}\left( 0 \right) }}{{j!}}} {\left({ - \varepsilon u} \right) ^j} + \frac{{{\psi ^{\left({r + 1} \right) }}\left({- \varepsilon \eta u} \right) }}{{\left({r + 1} \right) !}}{\left({ - \varepsilon u} \right) ^{r + 1}}$$

for $0< \eta < 1$. Using (16) in (15) and changing the order of integration we obtain:

17 $$\begin{aligned}&\left\langle {\widetilde{\left({\cos x - \sin x} \right) }\left({{\varphi_\varepsilon },x} \right) \cdot \widetilde{{\delta ^{\left( r \right) }}\left({{\varphi_\varepsilon },x} \right) },\psi \left( x \right) } \right\rangle \\ &\quad = \frac{{{{\left({ - 1} \right) }^{r + 1}}}}{{{\varepsilon ^r}}}{\sum \limits_{i = 0}^r {\frac{{\left({ - 1} \right) }}{{i!}}} ^{{a_i}}}\int \limits_{ - l}^l {{\varphi ^{\left( r \right) }}\left( u \right) \sum \limits_{j = 0}^r {\frac{{{\psi ^{\left( j \right) }}\left( 0 \right) }}{{j!}}} {{\left({ - \varepsilon u} \right) }^j}\int \limits_{ - l}^l {{{\left({\varepsilon t - \varepsilon u} \right) }^i}} \varphi \left( t \right) dt} du + O\left( \varepsilon \right) \\ &\quad =\frac{{{{\left({ - 1} \right) }^{r + 1}}}}{{{\varepsilon ^r}}}{\sum \limits_{i = 0}^r {\frac{{\left({ - 1} \right) }}{{i!}}} ^{{a_i}}}\sum \limits_{j = 0}^r {\frac{{{\psi ^{\left( j \right) }}\left( 0 \right) }}{{j!}}} {\left({ - \varepsilon } \right) ^j}\int \limits_{ - l}^l {{u^j}{\varphi ^{\left( r \right) }}\left( u \right) \int \limits_{ - l}^l {{{\left({\varepsilon t - \varepsilon u} \right) }^i}} \varphi \left( t \right) dt} du + O\left( \varepsilon \right) \\ &\quad = \sum \limits_{i,j = 0}^r {\frac{{{{\left({ - 1} \right) }^{r + 1 + {a_i} + j}}{\psi ^{\left( j \right) }}\left( 0 \right) }}{{i!j!{\varepsilon ^{r - j}}}}} \int \limits_{ - l}^l {{u^j}{\varphi ^{\left( r \right) }}\left( u \right) \int \limits_{ - l}^l {{{\left({\varepsilon t - \varepsilon u} \right) }^i}} \varphi \left( t \right) dt} du + O\left( \varepsilon \right) \\ &\quad =\sum \limits_{i,j = 0}^r {\frac{{{{\left({ - 1} \right) }^{r + 1 + {a_i} + j}}{\psi ^{\left( j \right) }}\left( 0 \right) }}{{i!j!{\varepsilon ^{r - j}}}}} {J_{i,\,j}} + O\left( \varepsilon \right) \end{aligned}$$

where $${J_{i,\,j}} = \int\limits_{ - l}^l {\varphi \left( t \right) dt\int\limits_{ - l}^l {{{\left({\varepsilon t - \varepsilon u} \right) }^i}} } {u^j}{\varphi ^{\left( r \right) }}\left( u \right) du$$; for $$i,\,j = 0,1,2,\ldots r$$.

Now using binomial expansion, for the last integral we obtain:

18 $$\begin{aligned} {J_{i,\,j}}&= \int \limits_{ - l}^l {\varphi \left( t \right) dt\int \limits_{ - l}^l {\left[ {\sum \limits_{k = 0}^i {\left( \begin{array}{l} i\\ k \end{array} \right) {{\left({\varepsilon t} \right) }^k}{{\left({ - \varepsilon u} \right) }^{i - k}}} } \right] } } {u^j}{\varphi ^{\left( r \right) }}\left( u \right) du \\&= \int \limits_{ - l}^l {\varphi \left( t \right) dt\sum \limits_{k = 0}^i {\left( \begin{array}{l} i\\ k \end{array} \right) {{\left({ - 1} \right) }^{i - k}}{\varepsilon ^i}} \int \limits_{ - l}^l {{t^k}} } {u^{i - k + j}}{\varphi ^{\left( r \right) }}\left( u \right) du \\&= \sum \limits_{k = 0}^i {\left( \begin{array}{l} i\\ k \end{array} \right) {{\left({ - 1} \right) }^{i - k}}{\varepsilon ^i}} \int \limits_{ - l}^l {{t^k}\varphi \left( t \right) dt\int \limits_{ - l}^l {{u^{i - k + j}}} } {\varphi ^{\left( r \right) }}\left( u \right) du \end{aligned}$$

The integral ${J_{a,b}} = \int\limits_{ - l}^l {{v^a}{\varphi ^{\left( b \right) }}\left( v \right) dv}$ is nonzero only for $a = b$ and its value is ${J_{a,a}} = {\left({ - 1} \right) ^a}a!$. Thus the only nonzero term in the above sum is obtained for $k = 0$ and $i+j=r$, and the value ${J_{i,j}}$ then will be

19 $${J_{i,\,j}} = {\left({ - 1} \right) ^i}{\varepsilon ^i} \cdot {\left({ - 1} \right) ^r}r! = {\left({ - 1} \right) ^{r + i}}{\varepsilon ^i}r!$$

Using $j = r - i$ and Eq. (19) in (17) we obtain:

20 $$\begin{aligned} &\left\langle {\left({\widetilde{\cos x - \sin x}} \right) \left({{\varphi_\varepsilon },x} \right) \cdot \widetilde{{\delta ^{\left( r \right) }}}\left({{\varphi_\varepsilon },x} \right) ,\psi \left( x \right) } \right\rangle \\ &\quad=\, \sum \limits_{i,j = 0}^r {\frac{{{{\left({ - 1} \right) }^{r + 1 + {a_i} + j}}{\psi ^{\left( j \right) }}\left( 0 \right) }}{{i!j!{\varepsilon ^{r - j}}}}\cdot } {\left({ - 1} \right) ^{r + i}}{\varepsilon ^i}r! + O\left( \varepsilon \right) \\ &\quad=\, \sum \limits_{i = 0}^r {\left({\begin{array}{l} r\\ i \end{array}} \right) {{\left({ - 1} \right) }^{1 + r + {a_i}}}{\psi ^{\left({r - i} \right) }}\left( 0 \right) } + O\left( \varepsilon \right)\\ &\quad=\, \sum \limits_{i = 0}^r {\left({\begin{array}{l} r\\ i \end{array}} \right) {{\left({ - 1} \right) }^{1 + i + {a_i}}}\left\langle {{\delta ^{\left({r - i} \right) }}\left( x \right) ,\psi \left( x \right) } \right\rangle } + O\left( \varepsilon \right) \end{aligned}$$

Putting ${b_i} = 1 + i + {a_i} = 1 + i + \left[ {\frac{{i + 1}}{2}} \right]$, having in mind that ${\left({ - 1} \right) ^{1 + i + \left[ {\frac{{i + 1}}{2}} \right] }} = {\left({ - 1} \right) ^{1 + \left[ {\frac{i}{2}} \right] }}$ and passing to the limit, as $\varepsilon \rightarrow 0$, we obtain (10), which proves the Theorem 1.

### **Theorem 2**

*The product of the generalized functions* $\widetilde{\left({\sin x + \cos x} \right) }$ *and* $\widetilde{{\delta ^{\left( r \right) }}\left( x \right) }$ *for* $r=0,1,2,\dots$ *in* ${\mathcal{G}}({\mathbf{R}})$ *admits associated distribution and it holds:*

21 $$\widetilde{\left({\sin x + \cos x} \right) } \cdot \widetilde{{\delta ^{\left( r \right) }}\left( x \right) } \approx \sum \limits_{i = 0}^r {\left( \begin{array}{l} r\\ i \end{array} \right) } {\left({ - 1} \right) ^{{b_i}}}{\delta ^{\left({r - i} \right) }}\left( x \right)$$

*where* $${b_i} = 1 + \left[ {\frac{{i + 1}}{2}} \right]$$.

### *Proof*

Applying Taylor’s Theorem for the functions $\sin x$ and $\cos x$ we have:

22 $$\sin x + \cos x = \sum \limits_{i = 0}^r {{{\left({ - 1} \right) }^{{a_i}}}} \cdot \frac{{{x^i}}}{{i!}} + {R_r}\left( x \right)$$

where $${a_i} = \left[ {\frac{i}{2}} \right]$$.

Now following the proof of the previous theorem, using the same steps, we obtain (21).

### **Theorem 3**

*The product of the generalized functions* $\widetilde{{e^x}}$ *and* $\widetilde{{\delta ^{\left( r \right) }}\left( x \right) }$ *for* $r=0,1,2,\dots$ *in* ${\mathcal{G}}({\mathbf{R}})$ *admits associated* *distribution and it holds:*

23 $$\widetilde{{e^x}}\cdot \widetilde{{\delta ^{\left( r \right) }}\left( x \right) } \approx \sum \limits_{i = 0}^r {\left({\begin{array}{*{20}{l}} r\\ i \end{array}} \right) } {\left({ - 1} \right) ^{1 + i}}{\delta ^{\left({r - i} \right) }}\left( x \right)$$

### *Proof*

Expanding function ${{e^x}}$ in a Taylor series we have:

24 $${e^x} = \sum \limits_{i = 0}^r {\frac{{{x^i}}}{{i!}}} + {R_r}\left( x \right)$$

Thus if we take ${a_i} = 0$ for $i = 0,1,2\ldots$ in the proof of the Theorem 1 we obtain (23).

### *Remark*

We should notice here that the products $\left({\cos x \pm \sin x} \right) \cdot {\delta ^{\left( r \right) }}\left( x \right)$ and ${e^x} \cdot {\delta ^{\left( r \right) }}\left( x \right)$ make sense even in the classical setting (Schwartz space of distributions), since multiplication of a classical distribution by a smooth function is valid operation in the Schwartz theory, but we have obtained here results in terms of associated distributions, which is a faithful generalization of the 'weak’ equality in ${\mathcal{G}}({\mathbf{R}})$. The results obtained in our theorems are associated with terms consisting only of the delta function and its derivatives. We will also notice that the product $\sin x \cdot \delta \left( x \right)$ is not equal to zero in ${\mathcal{G}}({\mathbf{R}})$ if we consider the 'strong’ equality, but it is zero in the sense of association, i.e. in the sense of 'weak’ equality in ${\mathcal{G}}({\mathbf{R}})$.

## Conclusion

We have evaluated some products of generalized functions, involving derivatives of the Dirac delta function, in Colombeau algebra in terms of associated distributions. This is significant because products of this type are very often used not only in physics, especially in quantum physics, but in other natural sciences and engineering, too, as we can see in the cited literature. Colombeau differential algebra of generalized functions contains the space of Schwartz distributions as a subspace, and the product of elements in it is generalization of the product of distributions, and thus all the results obtained in this way can be reformulated as regularized products in the classical distribution theory.
